# Notes and News

**Published:** 1899-06-17

**Authors:** 


### Notes and News.

(Collected and Communicated.)

### HOSPITAL MEETINGS.

Alexandra Hospital for Children with Hip Disease.—Festival Dinner at half-past seven, on Tuesday, June 20th, at the Hotel Cecil, the Duke of Cambridge in the chair.

Central London Throat and Ear Hospital, Gray's Inn Road, W.C.—Annual Meeting at half-past three p.m., on Tuesday, June 20th, at the Hospital.

Mr. James Orr, of Glasgow, has left amongst other bequests of smaller amount to medical charities, £2,500 to the Glasgow Royal Infirmary, £2,250 to the Glasgow Western Infirmary, and £2,000 to the Victoria Infirmary.

The annual distribution of diplomas and certificates by the National Health Society was patronised by a distinguished company. The Duke of Westminster presided, and the Princess Christian presented the diplomas, medals, and certificates to 103 successful candidates. The Bishop of London delivered an address, and moved a resolution in favour of the aims and methods of the society, which was seconded by Sir William Broadbent.

A new marine hospital is being established at New Mexico, to be used as a Consumption Sanatorium for the sailors of the merchant service in the United States. It is to be managed on the same lines as the National Home for Sailors in the United States, and will provide light employment for those of the inmates who desire it. Many acres of ground will be put under cultivation, and the patients will raise a considerable portion of the food consumed in the institution.

Hospital Sunday was marked by one or two special features this year. At Streatham the annual egg service was held, and upwards of 6,000 eggs were collected from the youthful congregation for the patients in the London hospitals; and in London the electrophone connected several hospitals with churches so that the patients might hear sermons preached on their behalf. The day was fine, and good collections are expected when the roll is published. There can be no doubt that the majority of the clergy do their utmost to interest their parishioners in the cause, and give all an opportunity of co-operating, whether they attend the services in the churches or not.

Mr. Arthur Gower has given £1,000 for the endowment of a bed in the Ipswich and East Suffolk Hospital.

Lord Pirbright has presented a site of five or six acres in extent for the erection of cottage homes of rest for discharged soldiers.

The new omnibus service between Waterloo Station and St. Thomas's Hospital with a halfpenny fare will benefit patients to the hospital.

The British Ophthalmic Hospital at Jerusalem has benefited to the extent of £310, the result of the Café Concert held in its aid at the Grafton Galleries.

The new buildings of the Royal Ophthalmic Hospital will be opened on Tuesday, June 27th, instead of Monday, June 26th, on which day the Royal Review at Aldershot will take place.

By the death of Dr. Norman Kerr the temperance cause will lose a zealous leader. Dr. Norman Kerr devoted his life to the study of inebriety, on which subject he wrote a large number of works.

Amongst the Fellows recently elected to the Royal Society are the following members of the medical profession: Dr. Henry Head, Assistant Physician at the London Hospital; Dr. E. Henry Starling; Dr. B. Coqhill Windle; and Major David Bruce, who is a Medical Graduate of Edinburgh University.

We regret to have to announce the death of Mr. Lawson Tait, of Birmingham. He had been in failing health for two or three months past, but his death took place rather unexpectedly on Tuesday last at his seaside home at Llandudno. Mr. Lawson Tait was educated at Heriot's Hospital and at the University of Edinburgh. He was a Fellow of the Royal Colleges of Surgeons of England and of Edinburgh, and was the possessor of many honorary degrees. He became house surgeon to the Clayton Hospital, Wakefield, in 1867, where he at once made his mark as a skilful surgeon and an ingenious operator. In 1871 he became connected with the Birmingham Hospital for Women, and from that time onwards devoted himself especially to abdominal surgery, a subject in regard to which he has continued to occupy a unique, if a somewhat combative, position. He was a trenchant writer, and possessed the art of using simple words in such a way as to give an appearance of common sense to much that he laid down. He was a man full of genius and of antipathies, principal among the latter of which must be placed Lister's antiseptic system and Spencer Wells' claim to fame in regard to ovariotomy. These were red rags; but in all other matters he brought to bear upon his work a mind full of originality and fingers apt at all the operative proceedings which he had introduced. Perhaps his work could not be better summarised than in a brief sentence which occurs in "Who's Who?" which, moreover, is somewhat characteristic of the man: "He has devoted himself to abdominal surgery, and has brought into existence numberless new operations for diseases of the abdomen, and has perfected and established many old ones; he is recognised all over the world as the pioneer of abdominal surgery, and his practice is co-extensive." In addition to his professional work Mr. Tait interested himself much in municipal and political affairs, and in 1886 he unsuccessfully contested one of the divisions of Birmingham as a Home Ruler.

